# Predicting Intensive Care Transfers and Other Unforeseen Events: Analytic Model Validation Study and Comparison to Existing Methods

**DOI:** 10.2196/25066

**Published:** 2021-04-21

**Authors:** Brandon C Cummings, Sardar Ansari, Jonathan R Motyka, Guan Wang, Richard P Medlin Jr, Steven L Kronick, Karandeep Singh, Pauline K Park, Lena M Napolitano, Robert P Dickson, Michael R Mathis, Michael W Sjoding, Andrew J Admon, Ross Blank, Jakob I McSparron, Kevin R Ward, Christopher E Gillies

**Affiliations:** 1 Michigan Center for Integrative Research in Critical Care Department of Emergency Medicine University of Michigan Ann Arbor, MI United States; 2 Department of Internal Medicine University of Michigan Ann Arbor, MI United States; 3 Department of Learning Health Sciences University of Michigan Ann Arbor, MI United States; 4 Michigan Institute for Data Science University of Michigan Ann Arbor, MI United States; 5 Department of Surgery University of Michigan Ann Arbor, MI United States; 6 Department of Microbiology & Immunology University of Michigan Ann Arbor, MI United States; 7 Department of Anesthesiology University of Michigan Ann Arbor, MI United States; 8 Department of Biomedical Engineering University of Michigan Ann Arbor, MI United States

**Keywords:** COVID-19, biomedical informatics, critical care, machine learning, deterioration, predictive analytics, informatics, prediction, intensive care unit, ICU, mortality

## Abstract

**Background:**

COVID-19 has led to an unprecedented strain on health care facilities across the United States. Accurately identifying patients at an increased risk of deterioration may help hospitals manage their resources while improving the quality of patient care. Here, we present the results of an analytical model, Predicting Intensive Care Transfers and Other Unforeseen Events (PICTURE), to identify patients at high risk for imminent intensive care unit transfer, respiratory failure, or death, with the intention to improve the prediction of deterioration due to COVID-19.

**Objective:**

This study aims to validate the PICTURE model’s ability to predict unexpected deterioration in general ward and COVID-19 patients, and to compare its performance with the Epic Deterioration Index (EDI), an existing model that has recently been assessed for use in patients with COVID-19.

**Methods:**

The PICTURE model was trained and validated on a cohort of hospitalized non–COVID-19 patients using electronic health record data from 2014 to 2018. It was then applied to two holdout test sets: non–COVID-19 patients from 2019 and patients testing positive for COVID-19 in 2020. PICTURE results were aligned to EDI and NEWS scores for head-to-head comparison via area under the receiver operating characteristic curve (AUROC) and area under the precision-recall curve. We compared the models’ ability to predict an adverse event (defined as intensive care unit transfer, mechanical ventilation use, or death). Shapley values were used to provide explanations for PICTURE predictions.

**Results:**

In non–COVID-19 general ward patients, PICTURE achieved an AUROC of 0.819 (95% CI 0.805-0.834) per observation, compared to the EDI’s AUROC of 0.763 (95% CI 0.746-0.781; n=21,740; *P*<.001). In patients testing positive for COVID-19, PICTURE again outperformed the EDI with an AUROC of 0.849 (95% CI 0.820-0.878) compared to the EDI’s AUROC of 0.803 (95% CI 0.772-0.838; n=607; *P*<.001). The most important variables influencing PICTURE predictions in the COVID-19 cohort were a rapid respiratory rate, a high level of oxygen support, low oxygen saturation, and impaired mental status (Glasgow Coma Scale).

**Conclusions:**

The PICTURE model is more accurate in predicting adverse patient outcomes for both general ward patients and COVID-19 positive patients in our cohorts compared to the EDI. The ability to consistently anticipate these events may be especially valuable when considering potential incipient waves of COVID-19 infections. The generalizability of the model will require testing in other health care systems for validation.

## Introduction

The effect of COVID-19 on the US health care system is difficult to overstate. It has led to unprecedented clinical strain in hospitals nationwide, prompting the proliferation of intensive care unit (ICU) capability and of lower-acuity field hospitals to accommodate the increased patient load. A predictive early warning system capable of identifying patients at increased risk of deterioration could assist hospitals in maintaining a high level of patient care while more efficiently distributing their thinly stretched resources. However, a recent review has illustrated that high quality validated models of deterioration in patients with COVID-19 are lacking [1]. All 16 of the models appraised in this review were rated at high or unclear risk of bias, mostly because of nonrepresentative selection of control patients. A primary concern is that these models may overfit to the small COVID-19 data sets that are currently available.

Early warning systems have been and continue to be applied in hospital settings prior to the COVID-19 pandemic to predict patient deterioration events before they occur, giving health care providers time to intervene [2]. The prediction of adverse events such as ICU admission and death provides crucial information to avert impending critical deterioration; it is estimated that 85% of such events are preceded by detectable changes in physiological signs [3] that may occur up to 48 hours before the event [4]. In addition, approximately 44% of events are avoidable through early intervention [5], and 90% of unplanned transfers to the ICU are preceded by a new or worsening condition [6,7]. Such abnormal signals indicate that predictive data analytics may be used to alert providers of incipient deterioration events, ultimately leading to improved care and reduced costs [8,9]. Given the number of unknowns surrounding the pathophysiology of COVID-19, early warning systems may play a pivotal role in treating patients and improving outcomes.

One model that has been assessed in patients with COVID-19 is the Epic Deterioration Index (EDI; Epic Systems Inc) [10,11]. The EDI is a proprietary clinical early warning system that aims to identify patients at an increased risk of deterioration and who may require a higher level of care. The EDI has the advantage over models built on COVID-19–specific data in that it is not overfit to small data sets, as it was trained on over 130,000 encounters [11,12]. Recent work has suggested it may be capable of stratifying patients with COVID-19 according to their risk of deterioration [11]. The outcomes used in this study were those considered most relevant to the care of patients with COVID-19 including ICU level of care, mechanical ventilation, and death. Although the EDI was able to successfully isolate groups of patients at very high and very low risk of deterioration, the overall performance as a continuous predictor was moderately low (area under the receiver operating characteristic curve [AUROC] 0.76, 95% CI 0.68-0.84; n=174) [11]. Additionally, much of the detail surrounding the EDI’s structure and internal validation has not been shared publicly. This makes the interpretation of individual predictions difficult. Since hospitals who do not use Epic electronic health record (EHR) systems may not have access to EDI predictions, we have also evaluated the publicly available National Early Warning Score (NEWS) as a secondary comparison.

In this study, we have applied our previously described model, Predicting Intensive Care Transfers and Other Unforeseen Events (PICTURE), to a cohort of patients testing positive for COVID-19 [13]. Initially developed to predict patient deterioration in the general wards, we have retrained the model to target those outcomes considered most relevant to the COVID-19 pandemic: ICU level of care, mechanical ventilation, and death. PICTURE, like the EDI, was trained and tuned on a large non–COVID-19 cohort including patients both with and without infectious diseases (131,546 encounters). Furthermore, we took extensive steps in the PICTURE framework to limit overfitting and learning missingness patterns in the data, such as a novel imputation mechanism [13]. This is critical in providing clinicians with novel, useful, and generalizable alerts, as missing patterns can vary in different settings and different patient phenotypes [13]. In addition to the risk score, PICTURE also provides actionable explanations for its predictions in the form of Shapley values, which may help clinicians easily interpret scores and better determine if actionability on the alert is required [14]. We validated this system in both a non–COVID-19 cohort and in patients who were hospitalized testing positive for COVID-19 and compared it to the EDI and NEWS on the same matched cohorts.

## Methods

### Setting and Study Population

The study protocol was approved by the University of Michigan’s Institutional Review Board (HUM00092309). EHR data was collected from a large tertiary, academic medical system (Michigan Medicine) from January 1, 2014, to November 11, 2020. The first 5 years of data (2014-2018; n=131,546 encounters) were used to train and validate the model, while 2019 data was reserved as a holdout test set (n=33,472 encounters). Training, validation, and test populations were segmented to prevent overlap of multiple hospital encounters between sets. Criteria for inclusion in these three cohorts were defined as 18 years or older and who were hospitalized (having inpatient or other observation status) in a general ward. We excluded patients who were discharged to hospice and whose ICU transfer was from a floor other than a general ward (eg, operating or interventional radiology unit) to exclude planned ICU transfers. We also excluded patients with a left ventricular assist device to avoid artifactual blood pressure readings.

To be included in the COVID-19 cohort (n=637 encounters), patients must have been admitted to the hospital with a COVID-19 diagnosis and have received a positive COVID-19 test from Michigan Medicine during their encounter. These patients were then filtered using the same criteria used in the 2019 test set, with the exception of the hospice distinction. Only discharged patients or those who already experienced an adverse event were included. Table 1 describes the study cohort and the frequency of individual adverse events. When compared to the non–COVID-19 test cohort from 2019, the proportion of Black and Asian patients was significantly higher (Black: 4214/33,472, 12.6% vs 220/637, 34.5%; *P*<.001; Asian: 686/33,472, 2.0% vs 29/637, 4.6%; *P*<.001). The rate of adverse events was also higher, rising from 4.0% (1337/33,472) to 24.3% (155/637; *P*<.001).

**Table 1:** Study population.^a^

Data set			Non–COVID-19							COVID-19			*P* value (non–COVID-19 vs COVID-19 test sets)^b^
			Training 2014-2018		Validation 2014-2018		Testing 2019		Testing 2020				
Encounters, n			105,457		26,089		33,472		637			N/A^c^	
Patients, n			62,392		15,597		23,368		600			N/A	
Age (years), median (IQR)			60.2 (46.5-70.8)		60.4 (46.7-71.2)		61.0 (47.0-71.5)		61.8 (49.6-72.0)			.02	
**Race, n (%)**													
	White	86,522 (82.0)		21,647 (83.0)		27,036 (80.8)		329 (51.6)			<.001		
	Black	12,344 (11.7)		2861 (11.0)		4214 (12.6)		220 (34.5)			<.001		
	Asian	2145 (2.0)		504 (1.9)		686 (2.0)		29 (4.6)			<.001		
	Other^d^	4446 (4.2)		1077 (4.1)		1536 (4.6)		59 (9.3)			<.001		
Female sex, n (%)			53,225 (50.5)		13,048 (50.0)		16,760 (50.1)		282 (44.3)			.003	
**Event rate^e^, n (%)**			4236 (4.0)		1007 (3.9)		1337 (4.0)		155 (24.3)			<.001	
	Death	920 (0.9)		232 (0.9)		277 (0.8)		16 (2.5)			<.001		
	ICU^f^ transfer	2979 (2.8)		717 (2.7)		1000 (3.0)		139 (21.8)			<.001		
	Mechanical ventilation	1330 (1.3)		299 (1.1)		352 (1.1)		49 (7.7)			<.001		
	Cardiac arrest^g^	143 (0.1)		37 (0.1)		56 (0.2)		N/A			N/A		

^a^Patients were subset into one of four study cohorts: a training set for learning model parameters, a validation set for model structure and hyperparameter tuning, a holdout test set for evaluation, and a final test set composed of patients testing positive for COVID-19. Values are based on individual hospital encounters.

^b^*P* values were calculated across the two test sets using a Mann-Whitney *U* test for continuous variables (age) and a chi-square test for categorical variables.

^c^N/A: not applicable.

^d^Other races comprising less than 1% of the population each were incorporated under the “Other” heading.

^e^The event rate represents a composite outcome indicating that one of the following events occurred: death, ICU transfer, mechanical ventilation, and cardiac arrest. The individual frequencies of these adverse events are also reported and represent the number of cases where each particular outcome was the first to occur. Please see the section Outcomes for the procedure of calculating these targets.

^f^ICU: intensive care unit.

^g^Cardiac arrest was not used as a target in the COVID-19 positive population, as the manually adjudicated data is not yet available at the time of writing.

### Predictors

The variables used as predictors were collected from the EHR and broadly included vital signs and physiologic observations, laboratory and metabolic values, and demographics. We selected specific features based on previous analysis [13]. Vital signs used in the model included heart rate, respiratory rate, pulse oximetry, Glasgow Coma Scale (GCS), urine output, and blood pressure. Laboratory and metabolic features included electrolyte concentrations, glucose and lactate, and blood cell counts. Demographics included age, height, weight, race, and gender. Fluid bolus and oxygen supplementation were also included as features. A full list of features is presented in Table S1 in Multimedia Appendix 1 alongside their respective median, IQR, and missingness rate. Variables centered on treatment (eg, medication administration) were largely excluded as, similar to the missingness flags described in Gillies et al [13], the scores generated by the model may be less generalizable and novel to the clinician as patterns of care change between diseases (eg, COVID-19) or institutions. Multimedia Appendix 1 Table S2 describes the effects of including medications as features in more detail.

### Outcomes

The primary outcomes in the training, validation, and non–COVID-19 test cohorts (data collected from 2014 through 2019) were death, cardiac arrest (as defined by the American Heart Association’s *Get With The Guidelines*), transfer to an ICU from a general ward or similar unit, or need for mechanical ventilation. Determination of ICU transfer was based on actual location or accommodation level. Outcomes in the COVID-19 positive cohort differed slightly in two respects. First, cardiac arrest information was not available at the time of writing and so was not included. Second, the emergency procedures undertaken by the hospital to accommodate the high volume of patients with COVID-19 led to the delivery of critical care in non-ICU settings. Thus, “ICU level of care” is used to denote patients who were treated by ICU staff or given ICU-level care but who may not have been physically housed in a bed previously demarcated as an ICU bed. This information is derived from the admission, discharge, and transfer table. Level of care was used to determine ICU transfer in patients with COVID-19 in addition to actual location. We discarded observations occurring 30 minutes before the first event or later to be consistent with other approaches [15]. For observation-level predictions, individual observations were labeled positive if they occurred within 24 hours of any of the aforementioned events and negative otherwise. We refer to these composite adverse events as the *outcome* or *target* throughout the text. These outcomes were designed to closely follow those of a recent analysis of the EDI at Michigan Medicine [11].

To verify the accuracy of our automatically generated labels, a clinician (author MRM) manually reviewed the patient charts for 20 encounters to determine whether the patient was infected with COVID-19, whether the recorded event truly took place, and whether the event was unplanned. To do so, we randomly sampled two encounters (one positive, the other negative if available) from each patient service with eight or more encounters to ensure the accuracy of the labels across all services. The result was a sample of 20 encounters, 11 of which were positive. The recorded event of interest for each encounter was reviewed by the clinician to determine whether the event took place and whether it was emergent (not planned). For the patients that were labeled as negative, the clinician reviewed the entire patient chart to ensure that no adverse events occurred during the encounter. The results indicate that all 20 patients were infected with COVID-19, all the labels and the event times were accurate, and all the events were unplanned. This provides evidence that the automatically generated outcomes accurately identify unplanned adverse events.

### PICTURE Model Development

**Figure 1:**
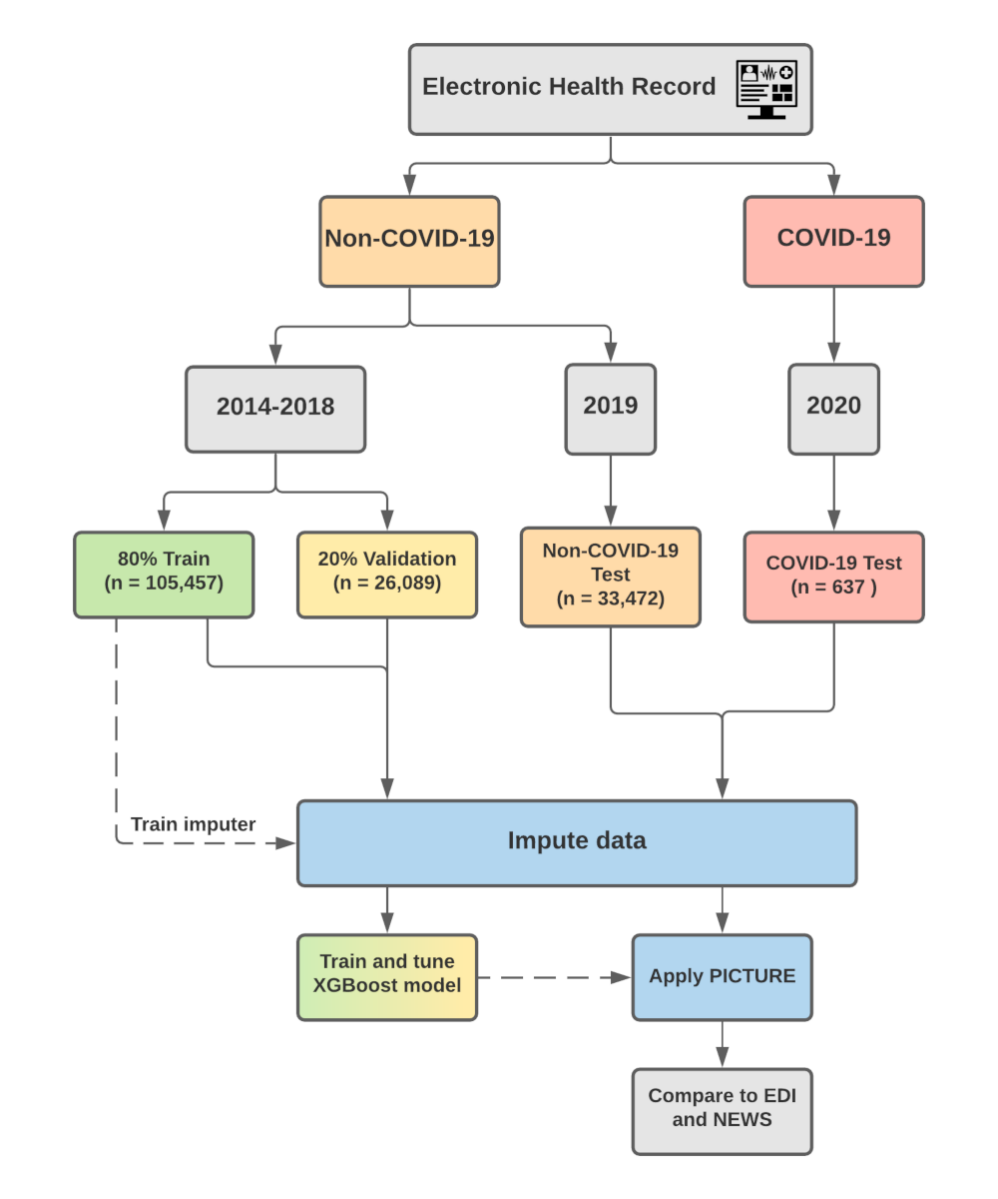
PICTURE training and validation framework. The electronic health record data is split into COVID-19 and non–COVID-19 patients. Encounters with an admission date between January 1, 2014, and December 31, 2018, were set aside for training (80%) and validation (20%) subsets. Encounters with an admission date between January 1 and December 31, 2019, were used as a non–COVID-19 test set. Encounters from 2020 that tested positive for COVID-19 were held out as a separate test set. In the case that a given patient has multiple encounters that overlap these boundaries, only the later encounters were considered to remove patient overlap between the cohorts. EDI: Epic Deterioration Index; NEWS: National Early Warning Score; PICTURE: Predicting Intensive Care Transfers and Other Unforeseen Events; XGBoost: extreme gradient boosting.

To train and evaluate the PICTURE model, we partitioned our data into four folds: a training and validation set using data from 2014 to 2018, a test set using 2019 data, and a fourth set consisting of data from patients who are COVID-19 positive. We partitioned the sets such that multiple hospital encounters from the same individual were restricted to one cohort, preventing patient-level overlap between cohorts. Encounters with an admission date from January 1, 2014, to December 31, 2018, were used for training and validation and hyperparameter tuning (n=131,546 encounters). These patients were further divided between training and validation sets using an 80%/20% split. Those patients with an admission date between January 1 and December 31, 2019, were reserved as a holdout test set (n=33,472 encounters). Lastly, patients testing positive for COVID-19 from March 1 to September 11, 2020, were reserved as a separate set (n=637 encounters). Figure 1 displays a graphical overview of this delineation.

As the EHR stores data in a long format (with each new row corresponding to a new measurement at a new time point), it was first converted to a wide structure such that each observation represented all features at a given time point for a given patient. The training and validation sets were grouped into 8-hour windows to ensure that each encounter would have the same amount of observations for the same amount of time in the hospital, avoiding emphasis on patients who get more frequent updates while training the model as described in Gillies et al [13]. The 2019 and COVID-19 test sets were left in a granular format, where each new observation represented the addition of new data (eg, an updated vital sign). Vital signs and laboratory values were forward filled such that each observation represented the most up-to-date information available as of that time, and the only time series–adjusted variables were oxygen supplementation, oxygen device use, and oxygen saturation as measured by pulse oximetry (SpO_2_), which were represented by the maximum (oxygen supplementation and device) or minimum (SpO_2_) over the previous 24 hours. Otherwise, each observation contained only the most up-to-date data available as of that time point and did not take historical values in to account. The remaining missing values were iteratively imputed using the mean of the posterior distribution from a multivariate Bayesian regression model. This method has previously been demonstrated to reduce the degree to which tree-based models learn missingness patterns to bolster performance [13]. Classification was achieved using an extreme gradient boosting model (v 0.90), an open-source implementation of a gradient-boosting tree framework that fits additional iterations using the errors of previous results [16]. The model uses a binary cross-entropy objective function with a maximum tree depth of three nodes, a learning rate of 0.05, no minimum loss reduction, uniform sampling with a subsample parameter of 0.6, and stopped when the validation area under the precision-recall curve (AUPRC) had not improved for 30 rounds. The model was applied to individual observations independently—that is, the model used the latest information available (via forward filling). In this sense, time dependance was not modeled aside from those aforementioned variables. All analyses were performed using Python 3.8.2 (Python Software Foundation).

### Epic Deterioration Index and NEWS

The EDI is a proprietary model developed by Epic Systems Corporation. Michigan Medicine uses Epic as its electronic medical record system and has access to the EDI tool. Similar to PICTURE, it uses clinical data that are commonly available in the EHR to make predictions regarding patient deterioration. It was trained using a similar composite outcome including death, ICU transfer, and resuscitation as adverse events [11]. It is calculated every 15 minutes. Specific details surrounding its structure, parameters, or training procedures have not been shared publicly.

NEWS, developed by the Royal College of Physicians, is a second index used to detect patients at an increased risk of deterioration event such as cardiac arrest, ICU transfer, and death [17,18]. In contrast to the EDI, which is based on a proprietary system, the basis of the NEWS score is openly available. NEWS scores were calculated based on the algorithm described in Smith et al [17]. The original NEWS was selected over the updated NEWS2 score due to evidence that its performance was found to be higher when predicting adverse events in patients at risk of respiratory failure [19].

### PICTURE Model Evaluation

#### Evaluation of PICTURE Performance in Non–COVID-19 Cohort

We first assessed the performance of the PICTURE model on all 33,472 encounters in the holdout test set comprising patients from 2019. Another early warning aggregate score, NEWS, was used for comparison in this preliminary analysis [17,18]. For each observation time point, the NEWS score was calculated according to their published scoring system and compared to PICTURE scores. Performance was assessed on two scales: observation level and encounter level. The term *observation level* is used to denote the performance of the model at each time the data for a patient is updated, with observations occurring 24 hours prior to a target event marked as 1 and otherwise marked as 0. Encounter level describes the model performance across the entire hospital encounter for one patient. It refers to the maximum model score during the patient’s stay, occurring between admission and at least 30 minutes (or longer for different minimal lead times; see the section Comparison of PICTURE to EDI in a Non–COVID-19 Cohort) before the first event. The target in this case is 1 if the patient ever met an outcome condition during their stay, and 0 otherwise.

#### Comparison of PICTURE and EDI

**Figure 2:**
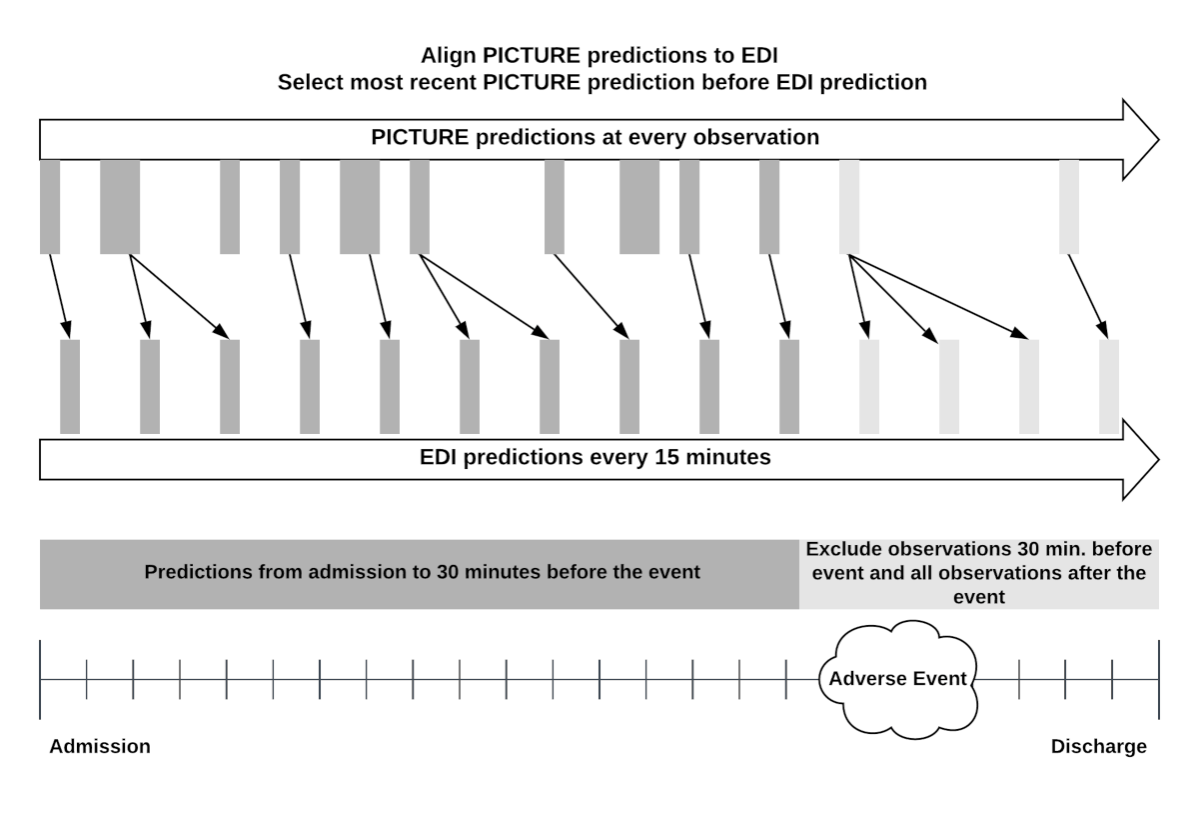
Alignment of PICTURE predictions to EDI scores. Although the PICTURE system outputs predictions each time a new observation (eg, a new vital sign) is input in to the system, the EDI score is generated every 15 minutes. To give the EDI any potential advantage, PICTURE scores are aligned to EDI scores by selecting the most recent PICTURE score before each EDI prediction. In both cases, observations occurring 30 minutes before the target and after are excluded (red). For the patients who did not experience an adverse event, the maximum score was calculated across the entire encounter. EDI: Epic Deterioration Index; PICTURE: Predicting Intensive Care Transfers and Other Unforeseen Events.

Since the EDI makes a prediction every 15 minutes, we simulated how the PICTURE score, calculated at irregular intervals each time a new data point arrives, would align with the EDI. This limited the available number of encounters to 21,740 in the 2019 test set and 607 encounters in the COVID-19 cohort. The PICTURE scores were merged onto EDI values by taking the most recent PICTURE prediction before the EDI prediction. This was to give the EDI any advantages in the alignment procedure. Figure 2 displays a visual schematic of this alignment. We then evaluated the two models using the same observation-level and encounter-level methods described in the previous section.

#### Performance Measures

AUROC and AUPRC were used as the primary criteria for comparison between the models. AUROC can be interpreted as the probability that two randomly chosen observations (one with a positive target, the other negative) are ranked in the correct order by the model prediction score. AUPRC describes the average positive predictive value (PPV) across the range of sensitivities. We also calculated 95% CIs for encounter-level statistics with a bootstrap method using 1000 replications to compute pivotal CIs. For observation-level statistics, block bootstrapping was used to ensure randomization between encounters and within the observations of an encounter. *P* values for AUROC differences were computed by counting the fraction of bootstrapped test statistics less than 0. If there were no simulations where the test statistic was greater than 0, the *P* value was recorded as *P*<.001.

#### Feature Ranking and Prediction Explanation

Despite the many benefits yielded by increasingly advanced machine learning models, use of these models in the medical field has lagged behind other fields. One contributing factor is their complexity, which make the resulting predictions difficult to interpret and in turn make it difficult to build clinician trust [20]. To better provide insight into the PICTURE predictions, tree-based Shapley values were calculated for each observation. Borrowed from game theory, Shapley values describe the relative contribution of a feature to the model’s prediction [14,21]. Positive values denote features that influenced the model toward a high prediction score (here indicating a higher likelihood of an adverse event), while negative values indicate the feature pushed the model toward a lower prediction score. The sum of the Shapley values across a single prediction plus the mean log-odds probability of the model is proportional to the log-odds of the prediction probability. Shapley values can be used to provide insight into individual model predictions or aggregated to visualize global variable importance.

#### Calibration and Alert Thresholds

Neither PICTURE nor the EDI are calibrated scores—that is, even though their output ranges from 0 to 1 (or 0 to 100 in the case of EDI), these values do not reflect a probability of deterioration [11]. Furthermore, both PICTURE and the EDI were trained on cohorts of non–COVID-19 patients. which have a much lower event rate and therefore may require a different alert threshold. A calibration curve depicting PICTURE and EDI score quantiles against calculated risk is used to demonstrate the deviation of PICTURE and EDI scores from an estimated probability. Several simulated PICTURE alarm thresholds are then examined, calculated by aligning them to the EDI threshold suggested in Singh et al [11] via sensitivity, specificity, PPV, and negative predictive value (NPV). The performance at these thresholds simulates when and how often a clinician would receive alerts. Data from an example patient is also highlighted to demonstrate how these alert thresholds and Shapley values may interact to provide actionable insights to clinicians.

## Results

### Validation of PICTURE Performance in a Non-COVID-19 Cohort

The ability of the PICTURE model to accurately predict the composite target was first assessed using the 33,472 encounters in the holdout test set from 2019. To provide a baseline for comparison, NEWS scores were calculated alongside each PICTURE prediction output. The observation-level and encounter-level AUROC and AUPRC are presented with 95% CIs in Table 2. The observation-level event rate can be interpreted as the fraction of individual observations during which an adverse event occurred within 24 hours, while the encounter-level event rate refers to the proportion of hospital encounters experiencing such an event. The difference in AUROC between PICTURE and NEWS was 0.068 (95% CI 0.058-0.078; *P*<.001) on the observation level and 0.064 (95% CI 0.055-0.073; *P*<.001) on the encounter level. The difference in AUPRC was similarly significant, at 0.041 (95% CI 0.031-0.050; *P*<.001) and 0.141 (95% CI 0.120-0.162; *P*<.001) on the observation and encounter levels, respectively.

**Table 2:** Evaluation of PICTURE (performance in a non–COVID-19 cohort).

Granularity and analytic			AUROC^a^ (95% CI^b^)		*P* value^c^ (AUROC)			AUPRC^d^ (95% CI)			*P* value (AUROC)			Event rate (%)	
**Observation**						<.001						<.001			1.01
	PICTURE^e^	0.821 (0.810-0.832)					0.099 (0.085-0.110)								
	NEWS^f,g^	0.753 (0.741-0.765)					0.058 (0.049-0.064)								
**Encounter (n=33,472)**						<.001						<.001			3.99
	PICTURE	0.846 (0.834-0.858)					0.326 (0.301-0.351)								
	NEWS	0.782 (0.768-0.795)					0.185 (0.165-0.203)								

^a^AUROC: area under the receiver operating characteristic curve.

^b^95% CIs were calculated using a block bootstrap with 1000 replicates. In the case of the observation level, this bootstrap was blocked on the encounter level.

^c^*P* values are calculated using the bootstrap method outlined in the section Performance Measures.

^d^AUPRC: area under the precision-recall curve.

^e^PICTURE: Predicting Intensive Care Transfers and Other Unforeseen Events.

^f^NEWS: National Early Warning Score.

^g^NEWS is used as a baseline for comparison.

### Comparison of PICTURE to EDI in a Non–COVID-19 Cohort

**Figure 3:**
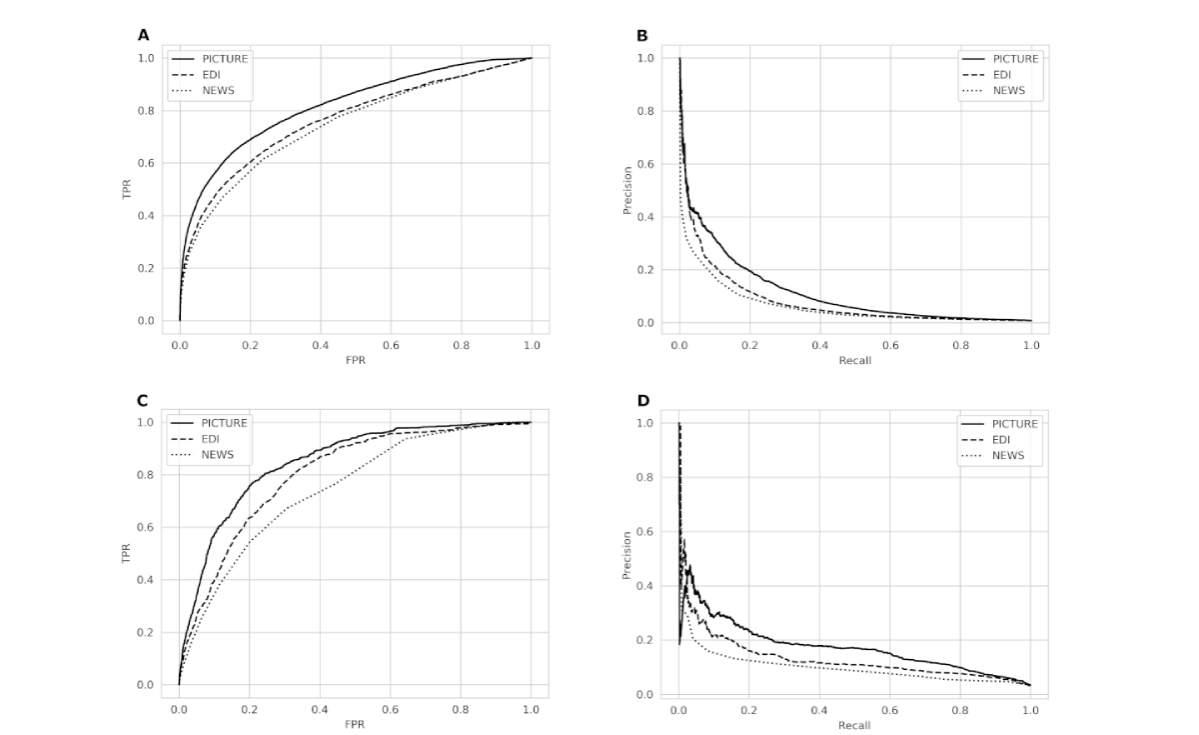
Comparison of PICTURE and the EDI. Panel A: receiver operating characteristic (ROC) curves for PICTURE, EDI, and NEWS models in the non–COVID-19 cohort. PICTURE area under the curve (AUC): 0.819; EDI AUC: 0.763; NEWS AUC: 0.745. Panel B: Precision-recall (PR) curves for the two models in the non–COVID-19 cohort. PICTURE AUC: 0.115; EDI AUC: 0.081; NEWS AUC: 0.062. Panel C: ROC curves for PICTURE, EDI, and NEWS models in the COVID-19 cohort. PICTURE AUC: 0.849; EDI AUC: 0.803; NEWS AUC: 0.746. Panel D: PR curves for the two models. PICTURE AUC: 0.173; EDI AUC: 0.131; NEWS AUC: 0.098 in the COVID-19 cohort. All curves represent observation-level analysis. EDI: Epic Deterioration Index; FPR: false-positive rate; NEWS: National Early Warning Score; PICTURE: Predicting Intensive Care Transfers and Other Unforeseen Events; TPR: true-positive rate.

PICTURE was then compared to the EDI model on non–COVID-19 patients in the same holdout test set from 2019. Due to limitations in available EDI scores, the number of encounters was restricted to 21,740. These time-matched scores were again evaluated using AUROC and AUPRC on the observation and encounter levels (Table 3). Panels A and B in Figure 3 display the associated receiver operating characteristic (ROC) and precision-recall (PR) curves for the observation-level performance. The difference in AUROC and AUPRC between PICTURE and the EDI reached significance on both the observation level (AUROC 0.056, 95% CI 0.044-0.068; *P*<.001; AUPRC 0.033, 95% CI 0.021-0.045; *P*<.001) and the encounter level (AUROC 0.056, 95% CI 0.046-0.065; *P*<.001; AUPRC 0.094, 95% CI 0.069-0.119; *P*<.001). NEWS results were similarly significant and are included in Table 3 for comparison.

**Table 3:** Comparison of PICTURE and the EDI in a non–COVID-19 cohort.

Granularity and analytic		AUROC^a^ (95% CI)	*P* value (AUROC)^b^	AUPRC^c^ (95% CI)	*P* value (AUPRC)	Event rate (%)	
**Observation**							0.77
	PICTURE^d^	0.819 (0.805-0.834)	vs EDI^e^: <.001 vs NEWS^f^: <.001	0.115 (0.096-0.130)	vs EDI: <.001 vs NEWS: <.001		
	EDI	0.763 (0.746-0.781)	vs NEWS: .01	0.081 (0.066-0.094)	vs NEWS: <.001		
	NEWS	0.745 (0.729-0.761)	N/A^g^	0.062 (0.051-0.072)	N/A		
**Encounter (n=21,740)**							4.21
	PICTURE	0.859 (0.846-0.873)	vs EDI: <.001 vs NEWS: <.001	0.368 (0.335-0.400)	vs EDI: <.001 vs NEWS: <.001		
	EDI	0.803 (0.788-0.821)	vs NEWS: .15	0.274 (0.244-0.301)	vs NEWS: <.001		
	NEWS	0.797 (0.781-0.814)	N/A	0.229 (0.204-0.254)	N/A		

^a^AUROC: area under the receiver operating characteristic curve.

^b^*P* values reflect the difference in AUROC or AUPRC.

^c^AUPRC: area under the precision-recall curve.

^d^PICTURE: Predicting Intensive Care Transfers and Other Unforeseen Events.

^e^EDI: Epic Deterioration Index.

^f^NEWS: National Early Warning Score.

^g^N/A: not applicable.

In addition to classification performance, lead time represents another critical component of a predictive analytics’ utility. Lead time refers to the amount of time between the alert and the actual event, and it determines how much time clinicians have to act on the model’s recommendations. We assessed the model’s relative performance at different lead times in a threshold-independent manner by excluding data occurring 0.5 hours, 1 hour, 2 hours, 6 hours, 12 hours, and 24 hours before an adverse event and calculating encounter-level performance (Table 4). In our cohort, PICTURE’s AUROC and AUPRC were significantly higher (*P*<.001) than the EDI model even when considering predictions made 24 hours or more before the actual event.

**Table 4:** Lead time analysis in non–COVID-19 cohort.^a^

Lead time (hours)	AUROC^b^ (95% CI)			AUPRC^c^ (95% CI)			Event rate (%)		Sample size, n
	PICTURE^d^	EDI^e^	PICTURE		EDI				
0.5	0.859 (0.846-0.873)	0.803 (0.787-0.820)	0.368 (0.336-0.400)		0.274 (0.244-0.302)	4.21		21,636	
1	0.850 (0.835-0.864)	0.795 (0.778-0.811)	0.346 (0.315-0.379)		0.254 (0.227-0.280)	4.18		21,636	
2	0.838 (0.823-0.853)	0.784 (0.767-0.802)	0.321 (0.292-0.352)		0.238 (0.210-0.265)	4.14		21,622	
6	0.825 (0.810-0.840)	0.768 (0.750-0.787)	0.280 (0.249-0.310)		0.210 (0.184-0.237)	3.92		21,572	
12	0.817 (0.801-0.832)	0.767 (0.749-0.786)	0.247 (0.215-0.275)		0.183 (0.159-0.207)	3.67		21,515	
24	0.808 (0.790-0.826)	0.759 (0.740-0.779)	0.205 (0.172-0.230)		0.144 (0.121-0.164)	3.24		21,419	

^a^The performance of the two models (encounter level) at various lead times were assessed by evaluating the maximum prediction score prior to *x* hours before the given event, with *x* ranging in progressively greater intervals from 0.5 to 24. On this cohort of non–COVID-19 patients, PICTURE consistently outperformed the EDI. At each level of censoring, the *P* value when comparing PICTURE to the EDI was <.001.

^b^AUROC: area under the receiver operating characteristic curve.

^c^AUPRC: area under the precision-recall curve.

^d^PICTURE: Predicting Intensive Care Transfers and Other Unforeseen Events.

^e^EDI: Epic Deterioration Index.

### Comparison of PICTURE to EDI in Patients With COVID-19

When applied to patients testing positive for COVID-19, PICTURE performed similarly well. PICTURE scores were again aligned to EDI scores using the process outlined in the section Comparison of PICTURE and EDI. This resulted in the inclusion of 607 encounters. Table 5 presents AUROC and AUPRC values for PICTURE and the EDI on both the observation and encounter levels with 95% CIs and includes NEWS scores for comparison. Panels C and D in Figure 3 display the associated ROC and PR curves. The difference in AUROC and AUPRC between PICTURE and the EDI reached statistical significance (α=5%) on the observation level (AUROC 0.046, 95% CI 0.021-0.069; *P*<.001; AUPRC 0.043, 95% CI 0.006-0.071; *P*=.002) and the encounter level (AUROC 0.093, 95% CI 0.066-0.118; *P*<.001; AUPRC 0.155, 95% CI 0.089-0.204; *P*<.001). Of note, the EDI results at the observation level (AUROC 0.803, 95% CI 0.771-0.838) were similar to those described in a previous validation (AUROC 0.76, 95% CI 0.68-0.84), although with a smaller confidence interval due to a larger sample size [11]. The differences in AUROC and AUPRC between PICTURE and NEWS also reached significance (α=5%) in patients with COVID-19, both on the observation level (AUROC 0.104, 95% CI 0.075-0.129; *P*<.001; AUPRC 0.076, 95% CI 0.033-0.105; *P*<.001) and the encounter level (AUROC 0.122, 95% CI 0.090-0.154; *P*<.001; AUPRC 0.224, 95% CI 0.151-0.290; *P*<.001).

As with the non–COVID-19 cohort, a similar lead time analysis was then performed to assess the performance of PICTURE and EDI when making predictions further in advance. Thresholds were again set at 0.5 hours, 1 hour, 2 hours, 6 hours, 12 hours, and 24 hours before the event, and observations occurring after this cutoff were excluded. In our cohort, PICTURE again outperformed the EDI even when making predictions 24 hours in advance (Table 6).

**Table 5:** Comparison of PICTURE and the EDI in patients testing positive for COVID-19.

Granularity and analytic		AUROC^a^ (95% CI)	*P* value (AUROC)	AUPRC^b^ (95% CI)	*P* value (AUPRC)	Event rate (%)	
**Observation**							3.20
	PICTURE^c^	0.849 (0.820-0.878)	vs EDI^d^: <.001 vs NEWS^e^: <.001	0.173 (0.116-0.211)	vs EDI: .002 vs NEWS: <.001		
	EDI	0.803 (0.772-0.838)	vs NEWS: <.001	0.131 (0.087-0.163)	vs NEWS: .002		
	NEWS	0.746 (0.708-0.783)	N/A^f^	0.098 (0.066-0.122)	N/A		
**Encounter (n=607)**							20.6
	PICTURE	0.895 (0.868-0.928)	vs EDI: <.001 vs NEWS: <.001	0.665 (0.590-0.743)	vs EDI: <.001 vs NEWS: <.001		
	EDI	0.802 (0.762-0.848)	vs NEWS: .05	0.510 (0.438-0.588)	vs NEWS: .02		
	NEWS	0.773 (0.732-0.818)	N/A	0.441 (0.364-0.510)	N/A		

^a^AUROC: area under the receiver operating characteristic curve.

^b^AUPRC: area under the precision-recall curve.

^c^PICTURE: Predicting Intensive Care Transfers and Other Unforeseen Events.

^d^EDI: Epic Deterioration Index.

^e^NEWS: National Early Warning Score.

^f^N/A: not applicable.

**Table 6:** Lead time analysis in COVID-19 cohort.^a^

Lead time (hours)	AUROC^b^ (95% CI)		AUPRC^c^ (95% CI)			Event rate (%)		Sample size, n
	PICTURE^d^	EDI^e^	PICTURE	EDI				
0.5	0.895 (0.867-0.926)	0.802 (0.761-0.842)	0.665 (0.586-0.739)	0.510 (0.436-0.587)	20.6		607	
1	0.887 (0.860-0.918)	0.793 (0.753-0.836)	0.631 (0.553-0.710)	0.491 (0.418-0.570)	20.5		606	
2	0.870 (0.840-0.901)	0.794 (0.754-0.833)	0.598 (0.518-0.675)	0.478 (0.400-0.555)	20.1		603	
6	0.847 (0.813-0.885)	0.769 (0.729-0.813)	0.552 (0.474-0.639)	0.435 (0.354-0.517)	19.3		597	
12	0.821 (0.783-0.863)	0.752 (0.708-0.798)	0.497 (0.411-0.577)	0.403 (0.333-0.480)	17.9		587	
24	0.808 (0.767-0.856)	0.740 (0.690-796)	0.443^f^ (0.344-0.529)	0.370 (0.289-0.459)	16.0		574	

^a^The performance of the two models (encounter level) at various lead times were again assessed by evaluating the maximum prediction score prior to x hours before the given event, with x ranging in progressively greater intervals from 0.5 to 24. On this cohort of non–COVID-19 patients, PICTURE consistently outperformed the EDI. At each level of censoring, the *P* value when comparing PICTURE to the EDI was <.001 unless otherwise marked.

^b^AUROC: area under the receiver operating characteristic curve.

^c^AUPRC: area under the precision-recall curve.

^d^PICTURE: Predicting Intensive Care Transfers and Other Unforeseen Events.

^e^EDI: Epic Deterioration Index.

^f^*P*=.001.

### Explanations of Predictions

**Figure 4:**
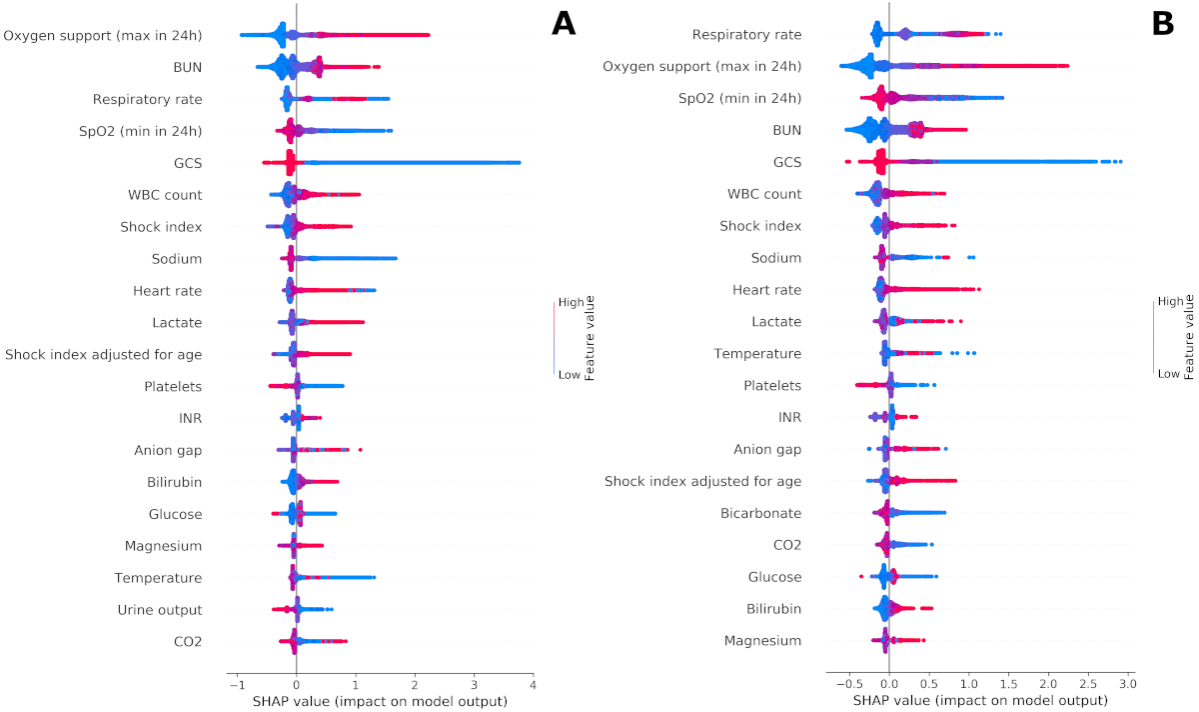
Shapley summary plots. Panel A depicts an aggregated summary plot of the Shapley values from the 2019 test set, while panel B corresponds to COVID-19 positive patients. The 20 most influential features are ranked from top to bottom, and the distribution of Shapley values across all predictions are plotted. The magnitude of the Shapley value is displayed on the horizontal axis, while the value of the feature itself is represented by color. For example, a large amount of oxygen support over 24 hours (red) in panel A was associated with a highly positive influence on the model, while low to no oxygen support (blue) pushed the model back toward 0. BUN: blood urea nitrogen; GCS: Glasgow Coma Scale; INR: international normalized ratio; SHAP: Shapley; WBC: white blood cells.

To provide clinicians with a description of factors influencing a given PICTURE score, we used Shapley values computed at each observation. Figure 4 depicts an aggregated summary of the 20 most influential features in the 2019 test set (panel A) and in the COVID-19 set (panel B). Positive Shapley values indicate that the variable pushed the PICTURE score toward a positive decision (ie, predicting an adverse event). Although many of the feature rankings appear similar between the 2019 and COVID-19 cohorts, we noted that respiratory variables such as respiratory rate, oxygen support, and SpO_2_ played a more pronounced role in predicting adverse events in COVID-19 positive patients than in non–COVID-19 patients. Multimedia Appendix 1 Figure S1 [22]. provides expanded detail on several of the variables (eg, respiratory rate and temperature) whose Shapley values do not appear to monotonically increase with their magnitude. One point of note is that the amount of oxygen support played a significant role in both cohorts. Although the EDI does not use the amount of oxygen support as a continuous variable, it does have a feature termed “oxygen requirement” [11]. To demonstrate that the observed improvement of PICTURE over the EDI is not driven solely by this additional information, oxygen support was binarized and the PICTURE model retrained. Although performance did decrease, indicating that the inclusion of oxygen support as a continuous variable is useful in predicting deterioration, PICTURE still outperformed the EDI on both the non–COVID-19 (difference in AUROC 0.057, AUPRC 0.082) and COVID-19 (difference in AUROC 0.035, AUPRC 0.050) cohorts. Thus, oxygen support alone does not account for the difference between PICTURE and EDI performance.

### Calibration and Alert Thresholds

Both PICTURE and the EDI return scores indicate a patient’s risk of deterioration; however, neither score is calibrated as a probability. Therefore, alert thresholds may provide a convenient mechanism to decide whether or not to alert a clinician that their patient is at increased risk. A previous study assessing the use of the EDI in patients with COVID-19 found that an EDI score of 64.8 or greater to be an actionable threshold to identify patients at increased risk [11]. As PICTURE scores lie on a different scale than the EDI, calibration is required to simulate PICTURE alert thresholds.

**Figure 5:**
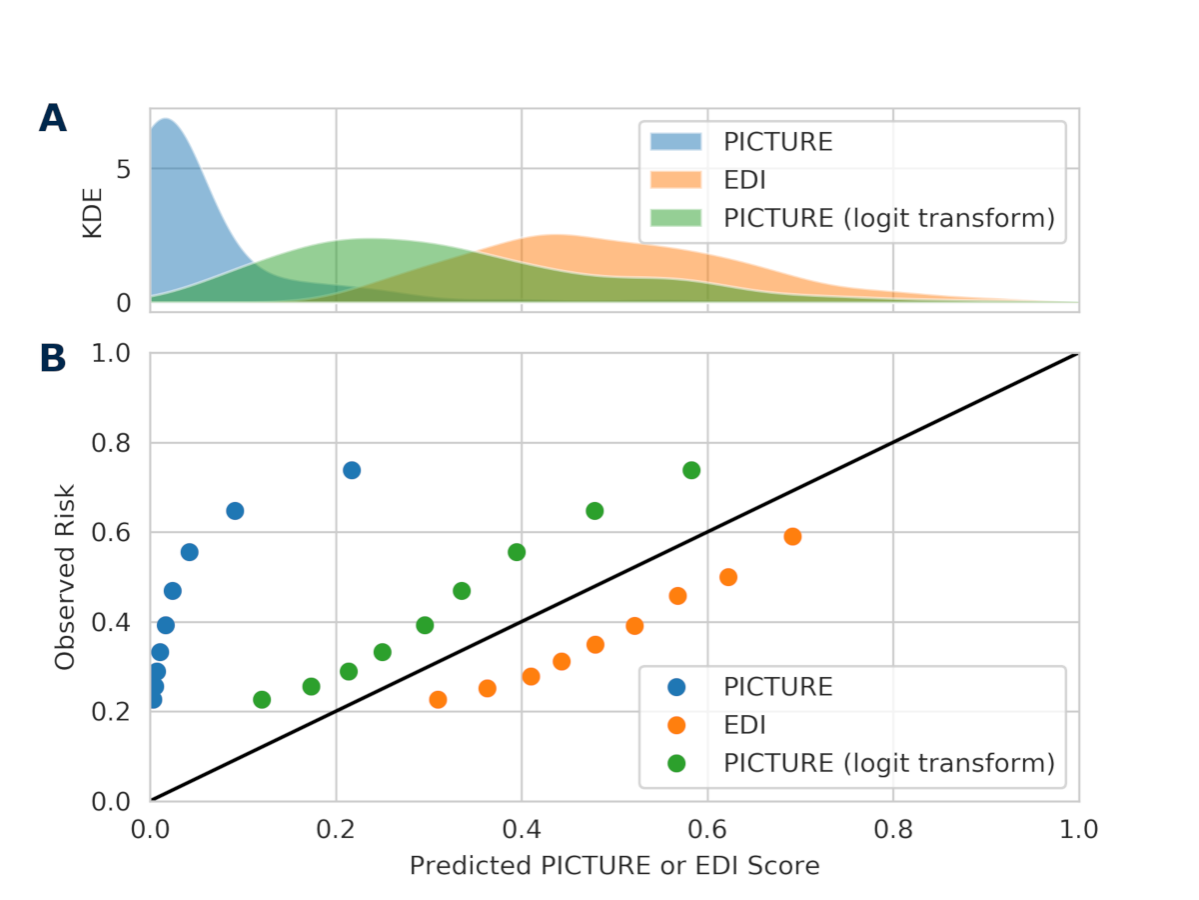
Distribution of scores and calibration curve. Panel A presents a KDE of the distribution of PICTURE and EDI scores. In addition to raw PICTURE scores, logit-transformed scores are also included. Panel B depicts quantiles of PICTURE and EDI scores (0.1, 0.2, 0.3,...0.9) against observed risk. Neither PICTURE nor the EDI are calibrated as probabilities, and as such, the use of set alarm thresholds may be useful to help alert clinicians when their patient is at an increased risk. EDI: Epic Deterioration Index; KDE: kernel density estimate; PICTURE: Predicting Intensive Care Transfers and Other Unforeseen Events.

Figure 5 depicts the distribution of PICTURE and EDI scores and a calibration curve comparing quantiles of PICTURE and EDI with observed risk. In this figure, EDI scores are rescaled from 0-100 to 0-1, while raw PICTURE scores are presented alongside a transformed score using a monotonically increasing function (logit transform) and scaled to the range 0-1. Based on this curve, the EDI appears to overestimate risk, while PICTURE may underestimate risk. However, neither metric is intended to reflect a probability. To more closely approximate a probability, techniques such as Platt scaling or isotonic regression may improve calibration in the future. Multimedia Appendix 1 Figure S2 illustrates the distribution of scores separated by positive and negative outcomes, and indicates that the PICTURE score may provide more separation between patients, something that the EDI has previously been demonstrated to struggle with [11].

To simulate when a clinician might receive an alert from the PICTURE system, four thresholds were selected, aligned based on the observed sensitivity, specificity, PPV, and NPV of the EDI score using the 64.8 value posed by Singh et al [11]. As an example, the *aligned by sensitivity* threshold listed in Table 7 was derived by determining the PICTURE threshold that had a sensitivity of 0.448, matching that of the EDI. Each of these thresholds, and their performances measured via F1 score, are compared to the EDI and are included in Table 7. The workup to detection ratio is calculated as 1 / PPV and indicates the number of false alerts a clinician might receive for each true positive [6]. For PICTURE, the workup to detection ratio ranged from 1.46 to 1.52 on the encounter level depending on the threshold used, compared to the EDI’s 1.71. The median time between alert and adverse event according to each threshold is also displayed. Confusion matrices describing the performance of the model at each threshold are included in Multimedia Appendix 1 (Table S3).

**Table 7:** Alert thresholds and median lead time.^a^

Score	Threshold source	Threshold value	Sensitivity	Specificity	PPV^b^	NPV^c^	WDR^d^	F1 score^e^	Lead time^f^ (h:min), median (IQR)
EDI^g^	Singh et al [11]	64.8	0.448	0.917	0.583	0.865	1.71	0.507	32:26 (4:37-66:08)
**PICTURE^h^**									
	Align by sensitivity	0.165	N/A^i^	0.946	0.683	0.869	1.46	0.541	40:14 (7:51-67:50)
	Align by specificity	0.097	0.616	N/A	0.658	0.902	1.52	0.636	40:04 (7:44-91:00)
	Align by PPV	0.048	0.792	0.851	N/A	0.940	N/A	0.668	54:10 (29:26-115:50)
	Align by NPV	0.173	0.432	0.946	0.675	N/A	1.48	0.527	41:40 (7:31-68:30)

^a^Sensitivity, specificity, PPV, and NPV were calculated for the EDI at a threshold of 64.8 as suggested in Singh et al [11] and based off encounter-level performance. PICTURE thresholds were then aligned to match these statistics. The WDR is also calculated as 1 / PPV and represents the number of false alarms received for each true positive. This value is important in limiting alert fatigue for clinicians and indicates that PICTURE may yield as much as 17% fewer false alarms for each true positive.

^b^PPV: positive predictive value.

^c^NPV: negative predicative value.

^d^WDR: workup to detection ratio.

^e^F1 scores were calculated as the harmonic mean between PPV and sensitivity.

^f^Lead times were determined using the intersection of true positives between PICTURE and the EDI, and were calculated as the time between a patient first crossing the threshold and their first deterioration event.

^g^EDI: Epic Deterioration Index.

^h^PICTURE: Predicting Intensive Care Transfers and Other Unforeseen Events.

^i^N/A: not applicable.

## Discussion

### Validation of PICTURE Performance in Non–COVID-19 Cohort

PICTURE makes a prediction at every observation for the features included. A natural starting point for the assessment of PICTURE’s performance is at this level of granularity. Using the general structure outlined in Gillies et al [13], we updated the PICTURE model to reflect the target outcomes of death, ICU transfer or accommodation, and mechanical ventilation within 24 hours. This updated model was tested on data from 33,472 encounters in 2019 to ensure its performance (observation-level AUROC 0.821) was reasonably consistent with that described in Gillies et al [13]. It was also compared to the NEWS scores at simultaneous time points and was found to have significantly outperformed NEWS (AUROC 0.753). These results confirm the findings in Gillies et al [13] using 2019 data instead of 2018 data. They also provide a baseline of comparison as we move to predictions made at uniform intervals instead of every observation.

### Comparison of PICTURE to EDI in a Non–COVID-19 Cohort

The EDI does not make predictions at every feature observation; instead, it makes predictions every 15 minutes. To provide a direct comparison to the EDI, we subset the PICTURE scores and time-matched them to the EDI scores as described in the section Performance Measures. PICTURE significantly outperformed the EDI on this cohort of non–COVID-19 patients, with an observation-level AUROC of 0.819 compared to the EDI’s AUROC of 0.763. This performance gap extended out over multiple lead times, and even when restricted to data collected 24 hours or more before the adverse event, PICTURE’s performance remained high with an AUROC of 0.808, while the EDI’s AUROC dropped to 0.759. These results suggest that using PICTURE, instead of the EDI, at the University of Michigan hospital will lead to less false alarms. PICTURE maintained the performance improvement even as the models were forced to make predictions with longer times before the adverse event.

### Comparison of PICTURE to EDI in Patients With COVID-19

As the EDI has increasingly been investigated as a feasible metric to gauge deterioration risk in patients with COVID-19 [11], we sought to apply our own deterioration model, PICTURE, to a cohort of patients with COVID-19. Although both models were trained and validated in non–COVID-19 general ward patients, their performance on our cohort of patients with COVID-19 was reasonably consistent with their respective results on our non–COVID-19 cohort. Even with a lower sample size (n=607 encounters), PICTURE significantly (*P*=.002) outperformed the EDI with an observation-level AUROC of 0.849 compared to the EDI’s AUROC of 0.803. PICTURE’s lead was again maintained 24 hours or more before the adverse event, with an AUROC of 0.808 versus the EDI’s AUROC of 0.740. These results suggest that using PICTURE instead of the EDI for patients with COVID-19 will lead to less alarm fatigue.

One important point of discussion is the considerably higher rate of deterioration observed in patients with COVID-19 (20.6% vs 4.21% of encounters). This is likely due to a combination of the severity of the virus when compared to a general ward population and the aggressive treatment regimen endorsed by clinicians facing a disease that, during the early phases of the pandemic, represented many unknowns. Therefore, the threshold selection presented in the section Calibration and Alert Thresholds may differ between COVID-19 and general ward patients. The performance of the PICTURE analytic (as measured by AUROC) increased slightly (though with overlapping 95% CIs) when applied to patients with COVID-19 versus the general test set, indicating that patients with COVID-19 may represent a slightly easier classification task. This is supported by the fact that the EDI also performed better on the COVID-19 cohort when measured by observation-level AUROC (0.763 vs 0.803), though this increase was not sustained in the encounter-level results (AUROC 0.803 vs 0.802).

### Explanations of Predictions

One key feature of the PICTURE model is its use of Shapley values to help explain individual predictions to clinicians. These explanations help add interpretability to the model, allowing clinicians to evaluate individual model scores and identify potential next steps, follow-up tests, or treatment plans. Figure 4 depicts an aggregated summary of Shapley values across all observations in both the COVID-19 and non–COVID-19 cohorts. In non–COVID-19 patients, a high degree of oxygen support, high blood urea nitrogen (BUN), very high or very low respiratory rate, low SpO_2_, and low GCS were the top five features most associated with high risk scores by the model. The COVID-19 cohort yielded the same top five features but reordered such that respiratory parameters (respiratory rate, oxygen support, and SpO_2_) ranked above BUN and GCS. Of note, temperature was one of the few features that changed direction between the two cohorts. In non–COVID-19 patients, a high temperature was associated with low to moderate risk, whereas high temperatures in patients with COVID-19 tended to indicate those with the highest risk scores. The aggregate feature explanations are, in general, similar between the two cohorts and are largely consistent with clinician intuition. However, these few key differences may reflect some of the unique challenges faced when caring for patients with COVID-19.

### Calibration and Alert Thresholds

Simulated alert thresholds were calculated based on the derived sensitivity, specificity, PPV, and NPV of the EDI threshold posited by Singh et al [11]. For each of the four thresholds, PICTURE outperformed the EDI according to the other four metrics as demonstrated in Table 7. For example, when the PICTURE alert threshold was adjusted such that its sensitivity matched the EDI’s (0.448); the specificity (0.946), PPV (0.683), and NPV (0.869) were all higher than the EDI’s (0.917, 0.583, and 0.865, respectively). Additionally, PICTURE’s workup to detection ratio ranged from 1.46 to 1.52 on the encounter level depending on the threshold used, compared to the EDI’s 1.71. This indicates that PICTURE may generate up to 17% fewer false positives for each true positive encounter.

### Case Study Example

**Figure 6:**
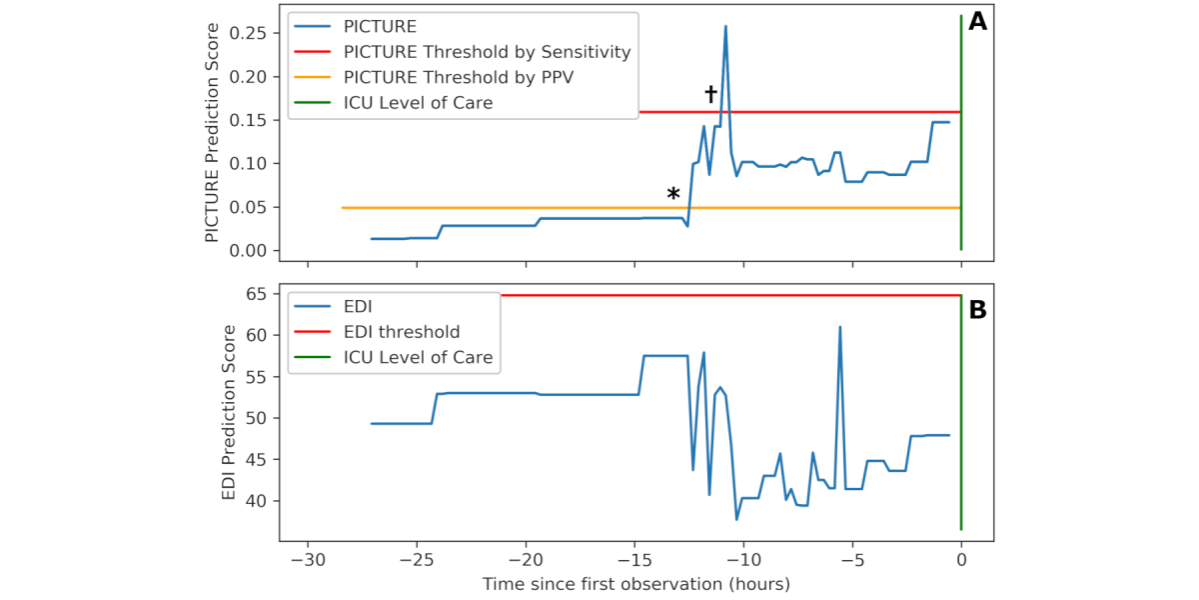
Sample trajectory of one patient. Panel A depicts the PICTURE predictions over 27 hours before the patient is eventually transferred to an ICU level of care (green bar). Two possible alert thresholds are noted: one (red: 0.165) based on the EDI’s sensitivity at a threshold of 64.8 (as suggested by Singh et al [11]), while the other (yellow: 0.048) is based on the EDI’s PPV at this threshold. Note that PICTURE peaks above the sensitivity-based threshold approximately 11 hours in advance of the ICU transfer and then remains elevated over the PPV threshold until the transfer occurs. * and † represent the first time points that PICTURE crossed each threshold, referenced in Table 7. Panel B demonstrates the EDI over the same time range, with the threshold of 64.8 suggested by Singh et al [11]. The EDI did not identify this patient as being at risk. EDI: Epic Deterioration Index; ICU: intensive care unit; PICTURE: Predicting Intensive Care Transfers and Other Unforeseen Events; PPV: positive predictive value.

As a demonstration of the potential utility of PICTURE, an individual hospital encounter was selected, and the trajectories of PICTURE and the EDI are visualized in Figure 6. The EDI score threshold of 64.8, suggested by Singh et al [11], and the sensitivity-aligned and PPV-aligned PICTURE thresholds are also depicted. Note that the PICTURE score remains low until approximately 12.5 hours before the adverse event (in this case, transfer to an ICU level of care), where it crosses the PPV-aligned threshold. Approximately 11 hours before the event, the PICTURE score peaks at a value of 0.235 and exceeds the sensitivity-aligned threshold of 0.165. After the initial peak, the PICTURE score then remains elevated, staying above the PPV-aligned threshold of 0.048 until the patient is transferred. In contrast, the EDI score never exceeded its alert threshold, and it dropped when the PICTURE score increased.

To simulate what a clinician receiving an alert from PICTURE might encounter, the Shapley values explaining the PICTURE predictions at both alert thresholds are recorded in Table 8. Note that these explanations are dominated by respiratory features, though heart rate and temperature are also present. Although these features may seem obvious in predicting the need for ICU care, it is worth highlighting that the EDI did not identify this patient as being at risk.

**Table 8:** Sample Predicting Intensive Care Transfers and Other Unforeseen Events explanations.

Rank and feature name^a^			Value		Median (IQR)^b^		Shapley score
**Shapley values after PPV ^c^ threshold (t – 12.75 h)**							
	1. Oxygen supplementation (rolling 24 h max)	7 L/min		2.0 (0.0-3.0)		1.06	
	2. SpO_2_^d^ (rolling 24 h min)	85%		92.0 (90.0-94.0)		0.93	
	3. Respiratory rate	26 bpm		20.0 (18.0-20.0)		0.76	
	4. Temperature	39.1 ˚C		36.9 (36.8-37.2)		0.32	
	5. Protein level	5.7		6.0 (5.6-6.4)		0.13	
**Shapley values after sensitivity threshold (t – 11** **h)**							
	1. Oxygen supplementation (rolling 24 h max)	35 L/min		2.0 (0.0-3.0)		1.93	
	2. SpO_2_ (rolling 24 h min)	85%		92.0 (90.0-94.0)		1.09	
	3. Respiratory rate	24 bpm		20.0 (18.0-20.0)		0.73	
	4. Heart rate^e^	124 bpm		83.0 (74.0-92.0)		0.71	
	5. Temperature	39.1˚C		36.9 (36.8-37.2)		0.32	

^a^The top 5 features corresponding to Predicting Intensive Care Transfers and Other Unforeseen Events predictions as it crosses the PPV-aligned threshold and the sensitivity-aligned threshold as noted in Figure 6. These predictions represent two possible locations where a clinician could receive an alert that their patient is deteriorating. Such information could be shared alongside the prediction score to provide better clinical utility to health care providers. Note that oxygenation (supplemental oxygen, SpO2, and respiratory rate) and temperature play a dominant role in both cases.

^b^The median and IQR are included for comparison, and are calculated using the COVID-19 data set.

^c^PPV: positive predictive value.

^d^SpO_2_: oxygen saturation as measured by pulse oximetry.

^e^Heart rate represented the primary difference between these two time points. When the Predicting Intensive Care Transfers and Other Unforeseen Events score first exceeded the PPV threshold 12.5 hours before the intensive care unit transfer, the heart rate remained at 65 bpm and was not among the top features as measured by Shapley. At 11 hours before the event, when the Predicting Intensive Care Transfers and Other Unforeseen Events score was at its highest, the heart rate had jumped to 124 bpm and was the fourth-most influential feature as measured by Shapley values.

### Limitations

This analysis is limited to a single academic medical center, and its generalizability to other health care systems will require future study. Our sample of patients with COVID-19 was also limited in size, limiting our power to detect differences between PICTURE and the EDI. Lastly, the thresholds presented in the section Calibration and Alert Thresholds may be different from those used in the general population due to the increased event rate. The thresholds may also require future tuning to suit the needs of individual units.

### Conclusion

The PICTURE early warning system accurately predicts adverse patient outcomes including ICU transfer, mechanical ventilation, and death at Michigan Medicine. The ability to consistently anticipate these events may be especially valuable when considering a potential impending second wave of COVID-19 infections. The EDI is a widespread deterioration model, which has recently been assessed in a COVID-19 population. Both PICTURE and the EDI were trained using approximately 130,000 non–COVID-19 encounters for general deterioration and thus are not overfit to the COVID-19 population [11,12]. Using a head-to-head comparison, we demonstrated that PICTURE has higher performance than the EDI at a statistically significant level (α=5%) for both COVID-19 positive and non–COVID-19 patients. In addition, PICTURE was capable of accurately predicting adverse events as far as 24 hours before the event occurred. Lastly, PICTURE has the ability to explain individual predictions to clinicians by displaying those variables that most influenced its prediction using Shapley values.

## Appendix

Multimedia Appendix 1 Supplementary material.

## Abbreviations

AUPRC: area under the precision-recall curve
AUROC: area under the receiver operating characteristic curve
BUN: blood urea nitrogen
EDI: Epic Deterioration Index
EHR: electronic health record
GCS: Glasgow Coma Scale
ICU: intensive care unit
NEWS: National Early Warning Score
NPV: negative predictive value
PICTURE: Predicting Intensive Care Transfers and Other Unforeseen Events
PODS: Propelling Original Data Science
PPV: positive predictive value
PR: precision-recall
ROC: receiver operating characteristic
SpO2: oxygen saturation as measured by pulse oximetry
