# Supplementary material for: Predicting Intensive Care Transfers and Other Unforeseen Events: Analytic Model Validation Study and Comparison to Existing Methods
Source: JMIR Med Inform. 2021 Apr 21;9(4):e25066. doi: 10.2196/25066 (PMC8061893; doi:10.2196/25066)
Supplement: Multimedia Appendix 1 [file medinform_v9i4e25066_app1.docx]

**Figure S1. Examination of Shapley values in individual variables.** One point of note when interpreting **(Figure 4)** is that in several of the key variables, the Shapley values do not change monotonically with variable magnitude. This can most clearly be seen with respiratory rate and temperature in Panel B of **(Figure 4),** representing COVID-19 patients. As is the case with many physiologic variables, features such as these lie within a clinically acceptable “normal range,” with deviations to either side often signaling wider physiologic issues. For example, a patient with a high temperature is said to be hyperthermic, while a low temperature indicates hypothermia – both are signs that the patient is not thermoregulating properly. Likewise, patients with extremely high or extremely low respiratory rates are likely struggling to oxygenate properly, an important indicator of deterioration regardless of the underlying disease state.


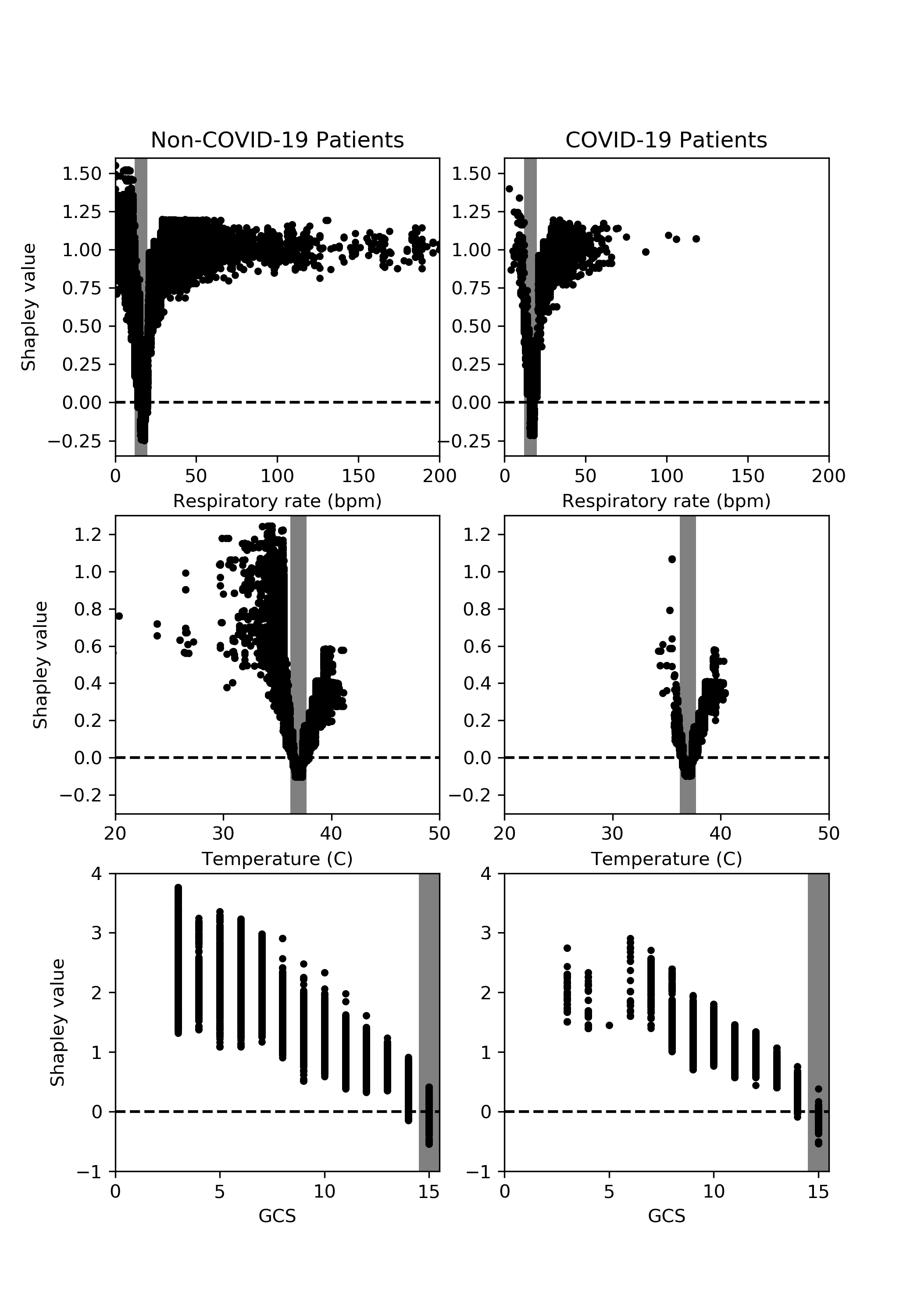
Figure S.1. demonstrates how the Shapley values change in relation to variable magnitude. The top two rows correspond to respiratory rate and temperature, respectively. Note that the Shapley values tend to increase in magnitude as these vital signs deviate from their respective normal ranges (indicated by gray shading; respiratory rate: 12-20 bpm, temperature 36.2 to 37.7˚C [22]), indicating that more extreme values push the model towards a more severe prediction. This behavior is contrasted with some features which do change monotonically, such as Glasgow Coma Score (GCS) (third row of panels). In this case, a “normal” score is also the highest possible score (15), and a score of 3 indicates no observable mental function. The relationship between GCS magnitude and the corresponding Shapley values approximates a monotonic decrease, indicating that lower mental status values push the model toward a more severe response.

**Figure S2. Distribution of PICTURE and EDI scores, separated by outcome labels.** Kernel density estimates are displayed for PICTURE (raw scores as well as logit-transformed) and the EDI. Distributions are separated by outcome label; that is, whether or not an adverse event occurred.

**
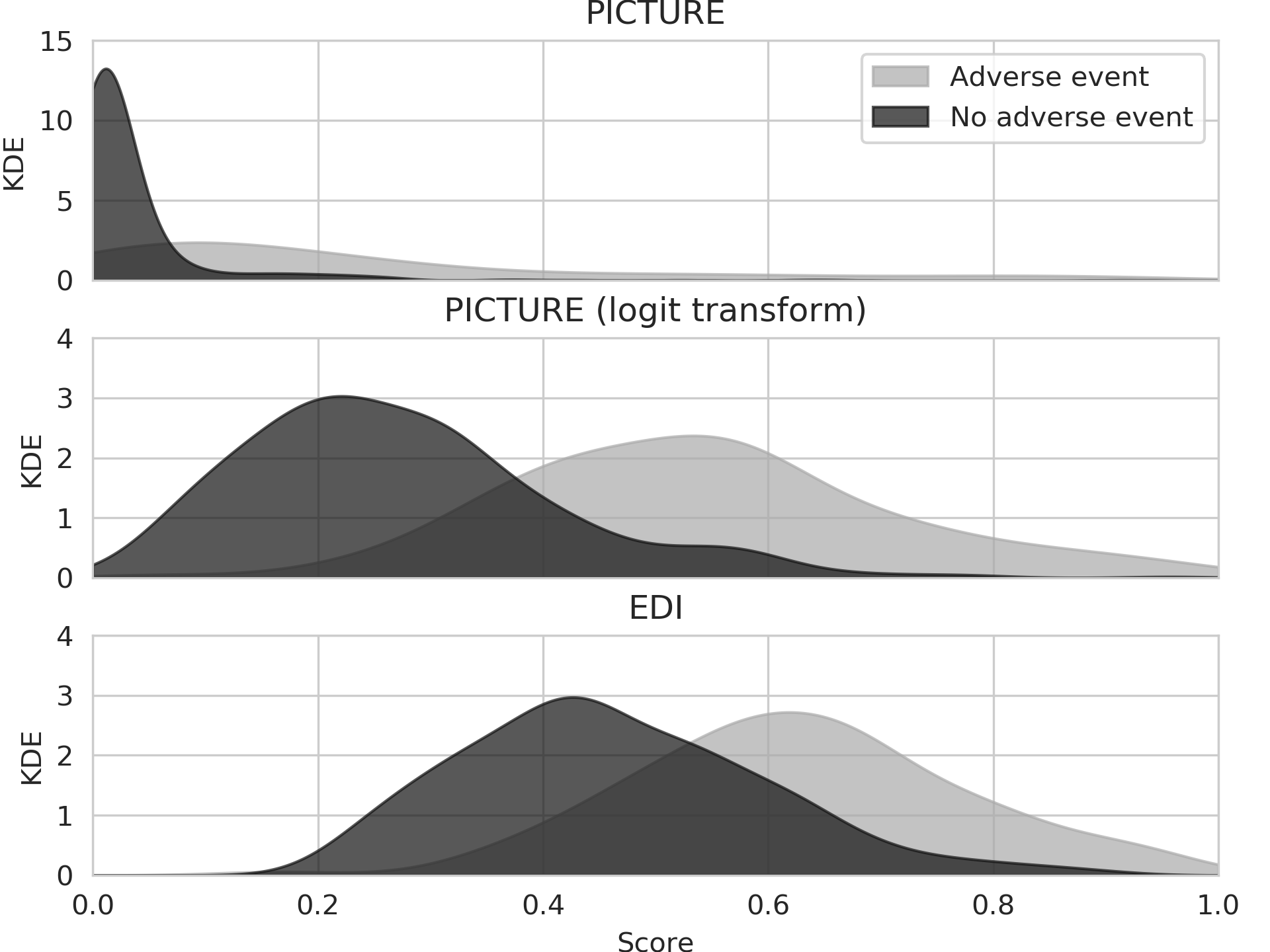
**

**Table S1. Feature list with descriptive statistics.** This table displays all features used in the PICTURE model along with their medians, interquartile range (IQR), and missing rate in all four cohorts. Values are computed on the encounter-level to avoid weighting the statistics by the number of observations per patient. Missingness rate represents the fraction of patients for whom there was no data available for the duration of their encounter. Gender and race are recorded as 1 if the patient meets the criteria (e.g. is female) and 0 otherwise. IV fluid bolus indicates a 1/0 flag if the patient received a fluid bolus during their stay. Oxygen supplementation and SpO_2_ values represent the maximum and minimum values over 24 hours, respectively. Abbreviations: BUN (blood urea nitrogen), GCS (Glasgow coma score), HGB (hemoglobin), INR (international normalized ratio), MAP (mean arterial pressure), MCH (mean corpuscular hemoglobin), MCHC (mean corpuscular hemoglobin concentration), MCV (mean corpuscular volume), MPV (mean platelet volume), PT (prothrombin time), PTT (partial thromboplastin time), RBC (red blood cell count), RDW (red cell distribution width), WBC (white blood cell count). * indicates that the feature was a binarized variable (e.g. did the patient receive IV fluids?) and the proportion of patients marked positive is recorded instead of median and IQR.

| **Feature Name** | **Training** | | | **Validation** | | | **Test** | | | **COVID-19** | | |
| --- | --- | --- | --- | --- | --- | --- | --- | --- | --- | --- | --- | --- |
|  | **(n=105,547 encounters)** | | | **(n = 26,089 encounters)** | | | **(n = 33,472 encounters)** | | | **(n = 475 encounters)** | | |
|  | **Median** | **IQR** | **Miss-ing** | **Median** | **IQR** | **Miss-ing** | **Median** | **IQR** | **Miss-ing** | **Median** | **IQR** | **Miss-ing** |
| **Age** | 60.2 | (46.5 - 70.8) | 0 | 60.4 | (46.7 - 71.2) | 0 | 61 | (47.0 - 71.5) | 0 | 61.8 | (49.6 – 72.0) | 0.00 |
| **Albumin** | 3.7 | (3.3 - 4.1) | 0.32 | 3.7 | (3.3 - 4.1) | 0.33 | 3.8 | (3.4 - 4.2) | 0.29 | 3.7 | (3.4 - 4.0) | 0.05 |
| **Anion Gap** | 11.7 | (10.2 - 13.2) | 0.01 | 11.7 | (10.3 - 13.2) | 0.01 | 11.7 | (10.3 - 13.3) | 0.01 | 12.7 | (11.5 - 14.1) | 0.02 |
| **Bicarb.** | 26 | (24.0 - 29.0) | 0.62 | 26 | (24.0 - 29.0) | 0.63 | 26 | (24.0 - 29.0) | 0.52 | 26 | (23.2 – 29.0) | 0.25 |
| **Bilirubin** | 0.5 | (0.3 - 0.9) | 0.34 | 0.5 | (0.3 - 0.8) | 0.35 | 0.5 | (0.4 - 0.9) | 0.3 | 0.5 | (0.4 - 0.8) | 0.05 |
| **BUN** | 16 | (11.0 - 23.0) | 0.01 | 16 | (11.0 - 22.5) | 0.01 | 17 | (12.0 - 24.0) | 0.01 | 17 | (12 - 25) | 0.03 |
| **Calcium** | 8.9 | (8.5 - 9.3) | 0.01 | 8.9 | (8.5 - 9.3) | 0.01 | 8.8 | (8.4 - 9.2) | 0.01 | 8.6 | (8.2 - 8.9) | 0.02 |
| **Chloride** | 105 | (102.0 - 107.0) | 0.01 | 105 | (102.0 - 107.0) | 0.01 | 105 | (102.0 - 107.0) | 0.01 | 103 | (100 - 106) | 0.01 |
| **CO_2_** | 26 | (24.0 - 28.0) | 0.01 | 26 | (24.0 - 28.0) | 0.01 | 26 | (24.0 - 28.0) | 0.01 | 26 | (24 - 28) | 0.02 |
| **Creatinine** | 0.8 | (0.7 - 1.1) | 0.01 | 0.8 | (0.7 - 1.1) | 0.01 | 0.9 | (0.7 - 1.1) | 0.01 | 0.9 | (0.7 - 1.2) | 0.02 |
| **Diastolic** | 67 | (61.0 - 74.0) | 0 | 67 | (61.5 - 74.0) | 0 | 67 | (62.0 - 74.0) | 0 | 69 | (64 - 75) | 0.00 |
| **Is Female*** | 0.505 | | 0 | 0.500 | | 0 | 0.501 | | 0 | 0.443 | | 0.00 |
| **GCS** | 15 | (15.0 - 15.0) | 0.2 | 15 | (15.0 - 15.0) | 0.2 | 15 | (15.0 - 15.0) | 0 | 15 | (15 - 15) | 0.03 |
| **Glucose** | 110 | (95.0 - 136.0) | 0 | 110 | (95.0 - 136.0) | 0 | 109 | (94.0 - 137.0) | 0 | 104 | (94.0 – 125.5) | 0.01 |
| **Height** | 67 | (64.0 - 70.0) | 0.13 | 67 | (64.0 - 70.0) | 0.13 | 67 | (64.0 - 70.0) | 0.11 | 67 | (64.0 - 70.0) | 0.16 |
| **Hematocrit** | 35 | (30.1 - 39.2) | 0.01 | 35.2 | (30.3 - 39.3) | 0.01 | 35.4 | (30.2 - 39.7) | 0 | 37.8 | (33.4 - 41.2) | 0.02 |
| **HGB** | 11.6 | (9.8 - 13.1) | 0.01 | 11.6 | (9.9 - 13.2) | 0.01 | 11.5 | (9.7 - 13.1) | 0.01 | 12.3 | (10.8 - 13.5) | 0.02 |
| **INR** | 1 | (1.0 - 1.2) | 0.42 | 1 | (1.0 - 1.2) | 0.42 | 1.1 | (1.0 - 1.2) | 0.43 | 1 | (1.0 - 1.1) | 0.45 |
| **IV Fluids*** | 0.680 | | 0 | 0.681 | | 0 | 0.589 | | 0 | 0.559 | | 0.00 |
| **Lactate** | 1.3 | (1.0 - 1.8) | 0.62 | 1.3 | (1.0 - 1.8) | 0.63 | 1.3 | (1.0 - 1.9) | 0.52 | 1.3 | (1.0 - 1.6) | 0.25 |
| **Magnesium** | 1.9 | (1.8 - 2.1) | 0.32 | 1.9 | (1.8 - 2.1) | 0.32 | 1.8 | (1.7 - 2.0) | 0.28 | 1.9 | (1.7 - 2.0) | 0.30 |
| **MAP** | 87.7 | (80.0 - 95.5) | 0 | 87.7 | (80.3 - 95.7) | 0 | 88 | (80.7 - 95.7) | 0 | 89 | (82 - 96) | 0.00 |
| **MCH** | 29.8 | (28.2 - 31.2) | 0.01 | 29.8 | (28.3 - 31.2) | 0.01 | 29.7 | (28.1 - 31.2) | 0.01 | 28.9 | (27.4 - 30.5) | 0.02 |
| **MCHC** | 33.2 | (32.3 - 34.1) | 0.01 | 33.2 | (32.3 - 34.1) | 0.01 | 32.6 | (31.7 - 33.5) | 0.01 | 32.5 | (31.7 - 33.5) | 0.02 |
| **MCHV** | 89.4 | (85.6 - 93.2) | 0.01 | 89.3 | (85.6 - 93.1) | 0.01 | 90.7 | (86.8 - 94.7) | 0.01 | 88.8 | (85.2 – 93.2) | 0.02 |
| **MPV** | 10.1 | (9.5 - 10.8) | 0.02 | 10.1 | (9.5 - 10.8) | 0.02 | 10.1 | (9.5 - 10.8) | 0.01 | 10.1 | (9.6 - 10.8) | 0.03 |
| **O_2_ Supp.** | 0 | (0.0 - 2.0) | 0 | 0 | (0.0 - 2.0) | 0 | 0 | (0.0 - 2.0) | 0 | 2 | (0.0 - 3.0) | 0.00 |
| **Phosphorus** | 3.4 | (3.0 - 4.0) | 0.43 | 3.4 | (3.0 - 4.0) | 0.43 | 3.5 | (3.0 - 4.1) | 0.43 | 3.3 | (2.8 - 3.8) | 0.50 |
| **Platelets** | 214 | (163.0-272.0) | 0.01 | 216 | (166.0-274.0) | 0.01 | 217 | (163.0-278.0) | 0.01 | 209 | (158 - 280) | 0.02 |
| **Potassium** | 4.2 | (3.9 - 4.5) | 0.01 | 4.2 | (3.9 - 4.5) | 0.01 | 4.2 | (3.9 - 4.4) | 0 | 4.1 | (3.8 - 4.4) | 0.01 |
| **Protein level** | 6.4 | (5.7 - 7.0) | 0.34 | 6.4 | (5.8 - 7.0) | 0.35 | 6 | (5.4 - 6.5) | 0.3 | 6 | (5.6 - 6.4) | 0.05 |
| **PT** | 10.9 | (10.3 - 12.2) | 0.43 | 10.9 | (10.3 - 12.0) | 0.43 | 10.9 | (10.3 - 12.0) | 0.44 | 10.7 | (10.3 - 11.5) | 0.46 |
| **PTT** | 26.4 | (24.3 - 29.6) | 0.52 | 26.4 | (24.3 - 29.5) | 0.52 | 25.2 | (23.4 - 28.0) | 0.53 | 26.4 | (24.3 - 29.3) | 0.52 |
| **Pulse** | 78.5 | (70.0 - 88.0) | 0 | 78 | (70.0 - 88.0) | 0 | 79 | (70.0 - 88.0) | 0 | 83 | (74 - 92) | 0.00 |
| **Pulse pressure** | 57 | (49 - 68) | 0 | 58 | (49 - 68) | 0 | 58 | (50 - 68) | 0 | 55 | (48 - 65) | 0.00 |
| **Is White*** | 0.82 | | 0 | 0.83 | | 0 | 0.808 | | 0 | 0.516 | | 0.00 |
| **Is Black*** | 0.117 | | 0 | 0.11 | | 0 | 0.126 | | 0 | 0.345 | | 0.00 |
| **Is Asian*** | 0.02 | | 0 | 0.019 | | 0 | 0.02 | | 0 | 0.046 | | 0.00 |
| **Is Other*** | 0.042 | | 0 | 0.041 | | 0 | 0.046 | | 0 | 0.093 | | 0.00 |
| **RBC** | 3.9 | (3.4 - 4.4) | 0.01 | 4 | (3.4 - 4.5) | 0.01 | 3.9 | (3.3 - 4.4) | 0.01 | 4.2 | (3.8 - 4.7) | 0.02 |
| **RDW** | 14 | (13.1 - 15.7) | 0.01 | 14 | (13.1 - 15.6) | 0.01 | 14.1 | (13.1 - 15.9) | 0.01 | 13.6 | (12.8 - 14.9) | 0.02 |
| **Resp. rate** | 18 | (16.0 - 18.0) | 0 | 18 | (16.0 - 18.0) | 0 | 17 | (16.0 - 18.0) | 0 | 20 | (18.0 - 20.0) | 0.00 |
| **Shock index** | 0.6 | (0.5 - 0.7) | 0 | 0.6 | (0.5 - 0.7) | 0 | 0.6 | (0.5 - 0.7) | 0 | 0.7 | (0.6 - 0.8) | 0.00 |
| **Shock index (age adjusted)** | 35.5 | (27.7 - 43.7) | 0 | 35.5 | (27.8 - 43.7) | 0 | 35.9 | (28.1 - 44.0) | 0 | 39.1 | (31.3 – 47.3) | 0.00 |
| **Sodium** | 139 | (137.0 - 141.0) | 0.01 | 139 | (137.0 - 141.0) | 0.01 | 139 | (137.0 - 140.0) | 0 | 138 | (136 - 140) | 0.01 |
| **SpO_2_** | 94 | (92.0 - 96.0) | 0 | 94 | (92.0 - 95.5) | 0 | 94 | (92.0 - 95.0) | 0 | 92 | (90 - 94) | 0.00 |
| **Systolic** | 126 | (115.0 - 139.0) | 0 | 127 | (115.0 - 139.0) | 0 | 127 | (116.0 - 140.0) | 0 | 127 | (116 - 138) | 0.00 |
| **Temp.** | 36.7 | (36.6 - 36.8) | 0 | 36.7 | (36.6 - 36.8) | 0 | 36.7 | (36.6 - 36.9) | 0 | 36.9 | (36.8 - 37.2) | 0.00 |
| **Urine output** | 250 | (150.0 - 400.0) | 0.12 | 250 | (150.0 - 400.0) | 0.12 | 250 | (150.0 - 380.0) | 0.15 | 200 | (100.0 - 350.0) | 0.22 |
| **Weight** | 178 | (147.2 - 214.5) | 0.02 | 178 | (147.0 - 215.0) | 0.02 | 179.2 | (147.5 - 216.3) | 0.01 | 187.0 | (158.9-228.7) | 0.04 |
| **WBC** | 8.2 | (6.1 - 10.7) | 0.01 | 8.2 | (6.2 - 10.8) | 0.01 | 8.1 | (6.1 - 10.7) | 0.01 | 6 | (4.7 - 8.2) | 0.02 |

**Table S2. Effects of including medications as features in the model.** As described in Section 2.B., variables regarding treatment or care of the patient were largely and intentionally excluded from the model. To demonstrate the effects of including such variables, a second model was developed which includes ordered and administered medications in the feature set. So as not to unduly dilute the pool of features, four classes of medications associated with deterioration were identified through clinician interview (AA). These include vasopressors (epinephrine, norepinephrine, dopamine, dobutamine, vasopressin, milrinone, or phenylephrine given intravenously), specific antibiotics (vancomycin, piperacillin, tazobactam, cefepime, meropenem, or aztreonam), diuretics (furosemide or bumetanide), and antiarrhythmics (amiodarone, digoxin, metoprolol, or diltiazem). Each observation was labeled with 1 if the patient was receiving a drug in the particular class at that time, and zero otherwise. The model was re-trained with these features, then applied to the 2019 (non-COVID-19) and COVID-19 test sets in an identical fashion to the primary model described above.

Our principle rationale for not including treatment variables such as medications was to help ensure generalizability. Similar to how missingness in vital signs or laboratory variables can falsely boost model performance [13], including medications as features may cause the model to learn institutional- or disease-specific patterns in physician behavior, which can reduce the ability of the model to generalize to other sources. For example, the relative prevalence of these medication categories in the 2019 and COVID-19 test sets are displayed in Panel A. Note that these features are truncated when the patient meets one of the target criteria (e.g. ICU transfer).

|  | **Vasopressors**  **(% encounters)** | **Antibiotics**  **(% encounters)** | **Diuretics**  **(% encounters)** | **Antiarrhythmics**  **(% encounters)** |
| --- | --- | --- | --- | --- |
| 2019 test set | 4.9% | 4.1% | 17.7% | 25.4% |
| COVID-19 test set | 1.3% | 0.9% | 14.1% | 17.9% |

The relative performance of PICTURE with and without the medication features is depicted in Panel B. While the inclusion of these medication features does slightly increase performance on the 2019 test set, this boost does not carry over to COVID-19 patients due in part to underlying difference in the frequency of medication usage.

| **Test set** | **Granularity** | **Analytic version** | **AUROC** | **AUPRC** | **Event Rate (%)** |
| --- | --- | --- | --- | --- | --- |
| **2019 (non-COVID-19)** | **Observation** | Original (no medications) | 0.819 | 0.115 | 0.77% |
|  |  | Including medications | 0.825 | 0.116 |  |
|  | **Encounter** | Original (no medications) | 0.859 | 0.368 | 4.21% |
|  |  | Including medications | 0.866 | 0.376 |  |
| **COVID-19** | **Observation** | Original (no medications) | 0.849 | 0.173 | 3.20% |
|  |  | Including medications | 0.847 | 0.171 |  |
|  | **Encounter** | Original (no medications) | 0.895 | 0.665 | 20.6% |
|  |  | Including medications | 0.895 | 0.696 |  |

Our second rationale was that including medications may decrease the utility and novelty of PICTURE alarms to a clinician. For example, if a patient is receiving a large number of medications and treatments such as vasopressors and antibiotics, the physician is likely already aware that the patient is struggling, and an alert reiterating this point interrupts their workflow, becoming redundant and possibly contributing to alert fatigue. Additionally, when using the alert thresholds outlined in Table 7, the median lead time in COVID-19 patients decreased by as much as two hours depending on the threshold used, giving clinicians less time to react to alerts. Therefore, as including treatment variables such as medications did not appear to significantly aid generalizability, lead time, or clinical utility, we removed these variables from the model.

**Table S3 Confusion matrices of predictions in COVID-19 test set.** Using the thresholds described in Table 7, four confusion matrices were constructed, with each cell containing the number and proportion of encounters (total *n* = 607 encounters). **Panel A** contains the number and proportion of true positives, true negatives, false positives, and false negatives for PICTURE using the threshold of 0.165, as aligned to the EDI by sensitivity. **Panel B** uses a threshold of 0.097, derived by matching specificity to the published EDI threshold. **Panel C** uses a threshold of 0.048 (aligned to the EDI via PPV). **Panel D** uses a threshold of 0.173 (aligned to the EDI by NPV). **Panel E** contains the EDI results using the published threshold of 64.8 [11].

| **Panel A: PICTURE Sensitivity-aligned threshold of 0.165** | | **Predicted** | |
| --- | --- | --- | --- |
|  |  | Positive | Negative |
| **Actual** | Positive | 56 (9.2%) | 69 (11.4%) |
|  | Negative | 26 (4.3%) | 456 (75.1%) |

| **Panel B: PICTURE Specificity-aligned threshold of 0.097** | | **Predicted** | |
| --- | --- | --- | --- |
|  |  | Positive | Negative |
| **Actual** | Positive | 77 (12.7%) | 48 (7.9%) |
|  | Negative | 40 (6.6% | 442 (72.9%) |

| **Panel C: PICTURE PPV-aligned threshold of 0.048** | | **Predicted** | |
| --- | --- | --- | --- |
|  |  | Positive | Negative |
| **Actual** | Positive | 99 (16.3%) | 26 (4.3%) |
|  | Negative | 72 (11.9% | 410 (67.5%) |

| **Panel D: PICTURE NPV-aligned threshold of 0.173** | | **Predicted** | |
| --- | --- | --- | --- |
|  |  | Positive | Negative |
| **Actual** | Positive | 54 (8.9%) | 71 (11.7%) |
|  | Negative | 26 (4.3%) | 456 (75.1%) |

| **Panel E: EDI at a threshold of 64.8** | | **Predicted** | |
| --- | --- | --- | --- |
|  |  | Positive | Negative |
| **Actual** | Positive | 56 (9.2%) | 69 (11.4%) |
|  | Negative | 40 (6.6%) | 442 (72.8%) |
